# Radiogenomic analysis of hypoxia pathway is predictive of overall survival in Glioblastoma

**DOI:** 10.1038/s41598-017-18310-0

**Published:** 2018-01-08

**Authors:** Niha Beig, Jay Patel, Prateek Prasanna, Virginia Hill, Amit Gupta, Ramon Correa, Kaustav Bera, Salendra Singh, Sasan Partovi, Vinay Varadan, Manmeet Ahluwalia, Anant Madabhushi, Pallavi Tiwari

**Affiliations:** 1 Case Western Reserve University, Department of Biomedical Engineering, Cleveland, 44106 USA; 2 Department of Neuroradiology, Imaging Institute, Cleveland Clinic, Cleveland, 44106 USA; 3 University Hospitals of Cleveland, Department of Radiology, Cleveland, 44106 USA; 4 Case Western Reserve University, School of Medicine, Cleveland, 44106 USA; 5 Brain Tumor and Neuro-Oncology Center, Cleveland Clinic, Cleveland, 44106 USA

## Abstract

Hypoxia, a characteristic trait of Glioblastoma (GBM), is known to cause resistance to chemo-radiation treatment and is linked with poor survival. There is hence an urgent need to non-invasively characterize tumor hypoxia to improve GBM management. We hypothesized that (a) radiomic texture descriptors can capture tumor heterogeneity manifested as a result of molecular variations in tumor hypoxia, on routine treatment naïve MRI, and (b) these imaging based texture surrogate markers of hypoxia can discriminate GBM patients as short-term (STS), mid-term (MTS), and long-term survivors (LTS). 115 studies (33 STS, 41 MTS, 41 LTS) with gadolinium-enhanced T1-weighted MRI (Gd-T1w) and T2-weighted (T2w) and FLAIR MRI protocols and the corresponding RNA sequences were obtained. After expert segmentation of necrotic, enhancing, and edematous/nonenhancing tumor regions for every study, 30 radiomic texture descriptors were extracted from every region across every MRI protocol. Using the expression profile of 21 hypoxia-associated genes, a hypoxia enrichment score (HES) was obtained for the training cohort of 85 cases. Mutual information score was used to identify a subset of radiomic features that were most informative of HES within 3-fold cross-validation to categorize studies as STS, MTS, and LTS. When validated on an additional cohort of 30 studies (11 STS, 9 MTS, 10 LTS), our results revealed that the most discriminative features of HES were also able to distinguish STS from LTS (*p* = 0.003).

## Introduction

Glioblastoma (GBM) is a highly aggressive malignant primary brain tumor with a median survival of 14 months^1^. Despite the well-established Stupp treatment protocol^2^ including surgical resection, radiotherapy plus concomitant and adjuvant temozolomide, the prognosis of GBM has only slightly improved (from 12 to 14 months) over the past two decades. A key pathway that drives tumor physiology towards treatment resistance and ultimately poor prognosis is tumor hypoxia, a by-product of abnormal tumor vasculature in aggressive GBM tumors. Tumor hypoxia is defined as the reduction in oxygen supply within a rapidly evolving GBM micro-environment. The need for more oxygen supply in hypoxic tumors activates hypoxia-inducible factor 1 alpha subunit (HIF1A) to produce vascular endothelial growth factor (VEGF) which in turn triggers angiogenesis to increase oxygen supply and sustain tumor’s survival and growth^3^. Unfortunately, hypoxia is known to be resistant to chemo-radiation, as it leads to an increase in the expression of (1) certain enzymes that are involved in resistance to temozolomide (a GBM chemotherapeutic agent), and (2) cancer stem cells that can withstand the effect of radiation. Recently, new anti-angiogenesis treatments such as small molecule inhibitors and VEGF inhibitors are being investigated as adjuvant treatment options for GBM tumors^4^. However, the clinical trials investigating these anti-angiogenic treatments have so far produced mixed results^5,6^. This is largely on account of enrolling “all-comers” to these trials, in the absence of tools to identify a subset of patients who will likely benefit from these anti-angiogenic treatments. Further, post anti-angiogenic treatments, there could be an amplification in pro-angiogenic factors such as Angiopoietin (ANG2) and decreased dependence on VEGF, which eventually may lead to higher extent of hypoxia, treatment resistance, and metastasis of GBM^7^. The ability to capture tumor hypoxia will have significant clinical implications towards personalized treatment options for GBM patients and improve our understanding of tumor behavior as well as patient’s outcome and response to specific treatments^8^.

As a standard-of-care protocol for brain tumor characterization, magnetic resonance imaging (MRI) is capable of capturing a diverse spectrum of tumor phenotypes. For instance, enhancement on Gd-T1w MRI is known to be correlated with blood brain barrier (BBB) disruption, while T2w/FLAIR abnormalities are known to capture proliferative tumor margins and vasogenic edema^9^. This suggests that the phenotypic differences at the cellular level are perhaps also reflected on MRI. Thus even though the visual appearance of different tumor phenotypes on MRI are similar, there nonetheless might be subtle sub-visual cues reflective of the differences in the micro-architectural appearance embedded in routine MRI that might enable distinction of molecularly distict GBM phenotypes. In this work, we hypothesized that phenotypic changes due to hypoxia-specific events (as observed on mRNA data), are also manifested on routinely acquired MRI (Gd-T1w, T2w, FLAIR); and can be captured using radiomic (high throughput computer extracted) features.

Recently, several studies have begun to explore the role of radiomic features on routine MRI scans (Gd-T1w, T2w, FLAIR) in capturing the underlying tumor pathology and molecular heterogeneity^10,11^. Radiomic features allow capture of quantitative imaging measurements by computing local macro- and micro-scale morphological changes in texture patterns (e.g. roughness, image homogeneity, regularity and edges) within the lesion. Many of these features, such as gray level co-occurrence matrix (GLCM)-based features, quantify enhancement heterogeneity, which has been shown to predict aggressive growth, unfavorable prognosis, and poor treatment response^12^. These texture-based radiomic MRI phenotypes have been shown to serve as surrogate markers for characterizing different molecular aberrations (including mutational status of IDH, 1p19q, MGMT) towards understanding GBM behavior and patient prognosis (also known as *radiogenomics*)^13–15^. However, to our knowledge, none of the existing studies have explored an explicit radiogenomic link between radiomic features obtained from routine MRI scans (i.e. Gd-T1w, T2w, FLAIR) and the hypoxia pathway and their role in predicting patient prognosis in GBM tumors. We present a radiogenomic approach to identify radiomic surrogate markers specific to the hypoxia pathway obtained from routinely acquired MRI scans (Gd-T1w, T2w, and FLAIR). We specifically focus on radiomic interrogation to capture the hypoxic events that lead to intratumoral molecular heterogeneity as manifested within the tumoral (necrosis, enhancing tumor) and peritumoral regions (non enhancing tumor and edema). Our work is motivated by previous studies that have demonstrated association of VEGF, a key mediator of tumor angiogenesis in the hypoxia pathway, with edema and tumor burden^16,17^.

In this work, we have two objectives. Firstly, we will identify a set of radiomic features obtained from Gd-T1w, T2w, FLAIR MRI scans, that were most discriminative of the extent of hypoxia in the tumor microenvironment, as measured using a hypoxia enrichment score (HES) using Single-sample Gene Set Enrichment Analysis (ssGSEA)^18^. Secondly, given that tumor hypoxia is known to have implications in GBM survival^6^, we seek to investigate the role of radiomic surrogate markers of hypoxia (as reflected on the HES), in discriminating patients’ overall survival (OS). The patients will be categorized based on their OS, as short-term survivors (OS < 7-months), mid-term survivors (7 months < OS < 16 months), and long-term survivors (OS > 16-months). Recent studies, including our own work, have independently investigated the use of radiomic descriptors in distinguishing short-term versus long-term survivors^12^. However, unlike previous studies, we present the first attempt at performing tumor and peritumoral interrogation towards (a) identifying radiomic surrogate markers of tumor hypoxia on pre-treatment MRI scans, and (b) evaluating their role in discriminating short-term, mid-term and long-term survivors of GBM. Our approach is intended to form a precursor to building novel image-based prognostic as well as predictive surrogate markers for personalizing treatment management in GBM, by reliably stratifying patients based on their hypoxia profile and overall survival.

The rest of the paper is organized as follows. Section 2 discusses the previous work and novel contributions. In Section 3, we provide methodological details of this work. Experimental results are presented in Section 4. We discuss the results in Section 5 and provide concluding remarks in Section 6.

## Previous work and Overview

Recently, there has been some work in identifying radio-genomic associations of hypoxia in GBM as well as other tumors. For instance, Yopp *et al* ^19^. have demonstrated inverse correlation of dynamic contrast-enhanced MR features with severity of hypoxia in hepatic cancers. Similarly, in a study by Diehn *et al*., proliferation and hypoxia gene expression patterns were found to be associated with the volume of mass effect and tumor contrast enhancement on different MRI protocols (Gd-T1w, T2w, FLAIR)^20^. However, to our knowledge, none of the works in the existing literature have attempted to establish a relationship between radiomic MR features obtained from different tumor sub-compartments and tumor hypoxia and then further employed these radiomic MRI surrogate markers of hypoxia as potential surrogate markers of overall survival in GBM.

**Figure 1:**
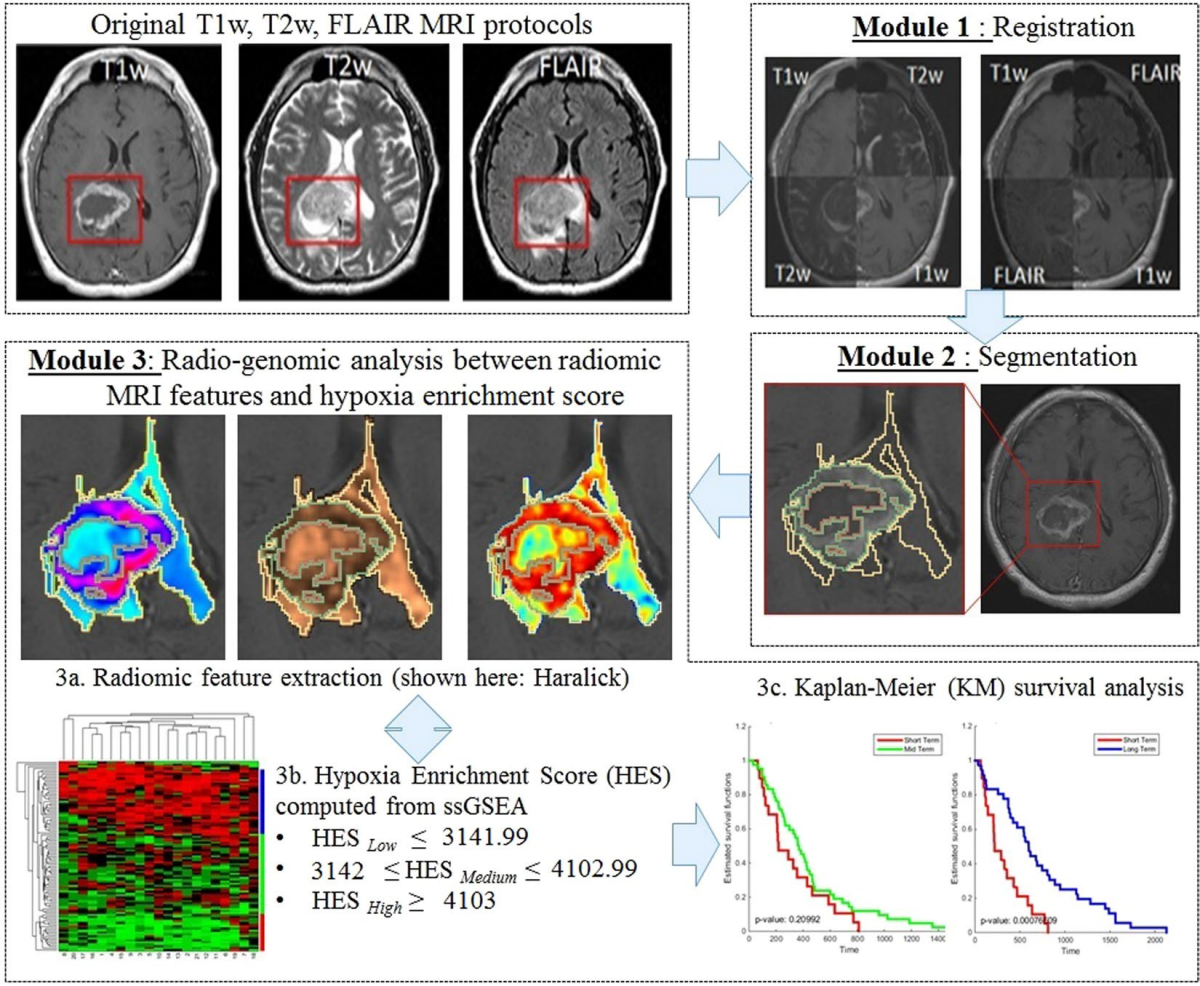
Overview of the methodology and overall work flow.

Figure 1 illustrates an overview of our framework. In Module 1, different MRI protocols are aligned in the same frame of reference; T2w, and FLAIR MRI were registered to Gd-T1w MRI in our case. In Module 2, the tumor sub-compartments including necrosis, edema, and enhancing tumor, are manually segmented by an expert using Gd-T1w, T2w, and FLAIR sequences, for each study. Module 3 involves computing radiomic features such as directional gradients (Gabor) and local intensity statistics (Haralick, Laws) from each of the tumor-specific sub-compartments (necrotic, enhancing tumor and peritumoral [edema and non enhancing tumor] regions) across the 3 MRI sequences. Within Module 3, top compartment-specific radiomic features are selected using mutual information^21^, based on their association with the hypoxia enrichment score, as obtained from the expression profile of 21 genes implicated in the hypoxia pathway (obtained via corresponding mRNA expression data)^20^. The top radiomic features that have the highest mutual association with hypoxia are then further employed to distinguish patients with short-term, mid-term, and long-term survival, using Kaplan-Meier survival analysis.

## Methods

### Study Population

Our cohort consisted of a total of 180 retrospectively analyzed treatment-naÃ¯ve multi-parametric MRI scans from the Cancer Imaging Archive (TCIA)^22^. TCIA is an open archive of cancer-specific medical images and associated clinical metadata which was curated by the National Cancer Institute (NCI) in association with participating institutions from the United States. Our inclusion criteria consisted of the following: (1) availability of all 3 routine MRI sequences (Gd-T1w, T2w, FLAIR) as well as RNA sequences for treatment-naïve patients, (2) MRI scans with diagnostic image quality (excluding the studies with image artifacts, as assessed by the expert reader), and (3) availability of individual overall survival information. A total of 65 cases were excluded either due to the absence of baseline scans, MRI artifacts, or unavailability of corresponding RNA sequence data or when one of the 3 MRI protocols (Gd-T1w, T2w, FLAIR) were not available. A total of 115 studies were used for further analysis. The corresponding RNA Seq for each of the 115 patients were collected from Broad Institute^23^. The 115 GBM subjects (69 males, age: 58.4 ± 12.56 yrs and 46 females, age: 56.47 ± 16.76 yrs), were then divided into three groups where the overall survival of the patients was stratified into short-term (≤7 months), long-term (>16 months)^24^, and the remaining into medium-term (>7 months to 16 months) survivors. Table 1 shows the patient demographics including mean overall survival, age, Karnofsky Performance Score (KPS) and gender of the population used in the study.

**Table 1:** Patient demographics of the study.

Cohort	Training Set			Independent Validation Set		
	Short Term	Mid Term	Long Term	Short Term	Mid Term	Long Term
Population (Patients)	22	32	31	11	9	10
Mean overall survival (in months)	3.9	11.4	29.8	4.2	11.69	27.7
Mean age (years)	64.5	60.3	53.2	57.8	55	49.6
Mean KPS	74	76	83	74	84	78
Gender	Male - 13	Male - 23	Male - 22	Male - 7	Male - 2	Male - 2
	Female - 9	Female - 9	Female - 9	Female - 4	Female- 7	Female -8

### Preprocessing

For every study, T2w and FLAIR were co-registered with reference to Gd-T1w MRI using 3D affine registration with 12 degrees of freedom encoding rotation, translation, sheer, and scale. The registration was performed using the General Registration (BRIANSFIT) module of 3D Slicer 4.5^25,26^. To resolve the issue of resolution variability, every MRI slice within a scan was re-sampled to have a uniform pixel spacing of 0.5 × 0.5 mm^2^ and was then interpolated to have 3 mm slice thickness. Skull stripping was done using the skull-stripping module in 3D Slicer^27^. Additional details regarding registration parameters employed in this work are provided in the Supplementary document. We then corrected the MRI protocols for known acquisition based intensity artifacts; bias field inhomogeneity and intensity non-standardness. Intensity non-standardness refers to the issue of MR image “intensity drift” across different imaging acquisitions. Intensity non-standardness results in MR image intensities lacking tissue-specific numeric meaning within the same MRI protocol, for the same body region, or for images of the same patient obtained on the same scanner. Intensity standardization was implemented in MATLAB R2014b (Mathworks, Natick, MA) using the method presented in Madabhushi *et al*.^28^, Another MRI artifact, bias-field inhomogeneity manifests as a smooth variation of signal intensity across the structural MRI, and has been shown to significantly affect computerized image analysis algorithms. Bias field artifacts were corrected for by means of the popular N4 bias-correction method^29^, which incrementally de-convolves smooth bias field estimates from acquired image data, resulting in a bias field corrected image.

### Segmentation

A total of three experts, each with over 6 years of experience in neuro-radiology were asked to perform the manual annotations on a total of 115 studies. Expert 1 (S.P, 6 years of experience) helped curate and manually annotate the training set. Expert 2 (V.H) with 7 years of experience in neuroradiology, manually annotated the validation set and was involved as one of the readers in the inter-observer variability experiments. Similarly, Expert 3 (A.G) who has over 8 years of radiology experience, independently annotated the validation set, and was involved as the second reader in the inter-observer variability experiments. Every 2-D slice of each MRI scan with visible tumor was manually annotated by the expert readers, into 3 regions (1) edema (which included the non enhancing tumor as well), (2) tumor necrosis, and (3) enhancing tumor. On Gd-T1w images, necrosis is relatively represented as hypointense regions which are commonly located in the central region of the tumor. Similarly, hyperintense FLAIR signals correlate with greater interstitial leakage and low cellular density, reflecting edema. Therefore, T2w and FLAIR scans were used to identify edema and necrosis and enhancing tumour was delineated based on Gd-T1w MRI.

To evaluate the effect of inter-observer variability in contouring the tumor sub-compartments, two experts were asked to independently manually segment 20 randomly chosen cases of GBM from our cohort (6 long term cases, 7 mid-term cases and 7 short term cases). Both the radiologists were provided separately with treatment naïve scans of Gd-T1w, T2w and FLAIR protocols. We obtained an average Dice Similarity Coefficient (DSC) scores across the 20 cases from the enhancing and edematous/nonenhancing region to be over 0.80.

### Compartment-specific radiomic feature extraction from MRI scans

A total of 30 2D radiomic features were extracted individually from every sub-compartment (edema/nonenhancing tumor, necrosis, enhancing tumor) for each of the 3 MR protocols (Gd-T1w, T2w, FLAIR). This resulted in a total of 270 features extracted for every study. The feature set for every study included 5 Laws energy, 12 Gabor, and 13 Haralick features on a per-pixel basis. A median feature value was then calculated from the feature responses of all pixels within the region of interest. All feature calculations were performed using in-house software implemented in MATLAB R2014b platform. A brief description of the extracted radiomic features is as follows:

- *Laws energy* (*5 descriptors*): Laws energy features use 5 × 5 window masks that are symmetric or anti-symmetric to extract level (L), edge (E), spot (S), wave (W), and ripple (R) patterns. These patterns are used to detect various types of textures on an image^30^.
- *Gabor energy* (*12 descriptors*): Gabor operators are the steerable class of gradients which attempts to match localized frequency characteristics^31^. A Gabor filter can be defined as the modulation of a complex sinusoid by a Gaussian function. Each descriptor quantifies response to a given Gabor filter at a specific frequency (*f* = 0, 4, or 16) and orientation (*θ* = 45°, 90°, 135°, 180°), and attempts to capture the prominent direction in which intensity changes occur^31^.
- *Haralick energy* (*13 descriptors*): Haralick texture features are based on quantifying the spatial gray-level co-occurrence within local neighborhoods around each pixel in an image^32^. These features potentially capture the structural heterogeneity within the region of interest. A total of 13 Haralick texture descriptors were calculated based on statistics derived from the corresponding co-occurrence matrices.

Detailed description of the set of features employed in this work and its possible relationship to the pathophysiology of GBM is provided in Table 2 below. The complete list of features extracted has been provided in the Supplementary spreadsheet (Sheet 1).

**Table 2:** Pathophysiological significance of radiomic features which possibly reflect biological traits of GBM and can be captured on MRI.

Feature category	Descriptor	Intuitive description	Relevance to GBM pathophysiology
Laws features	E5, L5, S5, R5 (combination in both X and Y directions)	E- Edges, L- Level, S- Spots, R- Ripples	Accounting for characteristic qualitative appearance of wave, ripple, edge and spots within an ROI
Gabor features	frequency (0, 4, or 16) and orientation (45°, 90°, 135°, 180°)	This filter bank has characteristics of spatial locality and orientation selectivity	Captures the prominent direction in which the intensity changes occur
Haralick features	Inverse difference moment (IDM)	IDM is a reflection of the presence or absence of uniformity, and hence is a measure of local regions of homogeneity High IDM: Higher presence of locally uniform windows in GLCM. Low IDM: Higher presence of locally heterogeneous windows in GLCM	Captures the underlying lesion heterogeneity
	Correlation	Quantifies the linear patterns in an image based on the distance parameter.	Increased presence of linear patterns yield higher correlation values, lack of image linearity yield lower correlation values
	Sum Entropy	Measure of GLCM relationship to distribution of intensity with respect to entropy (measure of disorder)	Higher entropy is indicative of more chaotic arrangement in areas of high viable cell population
	Sum Variance	Measure of GLCM relationship to distribution of intensity with respect to variance. High sum variance: greater standard deviation of sum average. Low sum variance: low standard deviation of sum average	Possibly accounting for greater variation of scattered atypia and local accumulation of mitotic processes as observed on histopathology.

### Generating Hypoxia Enrichment Score (HES)

**Figure 2:**
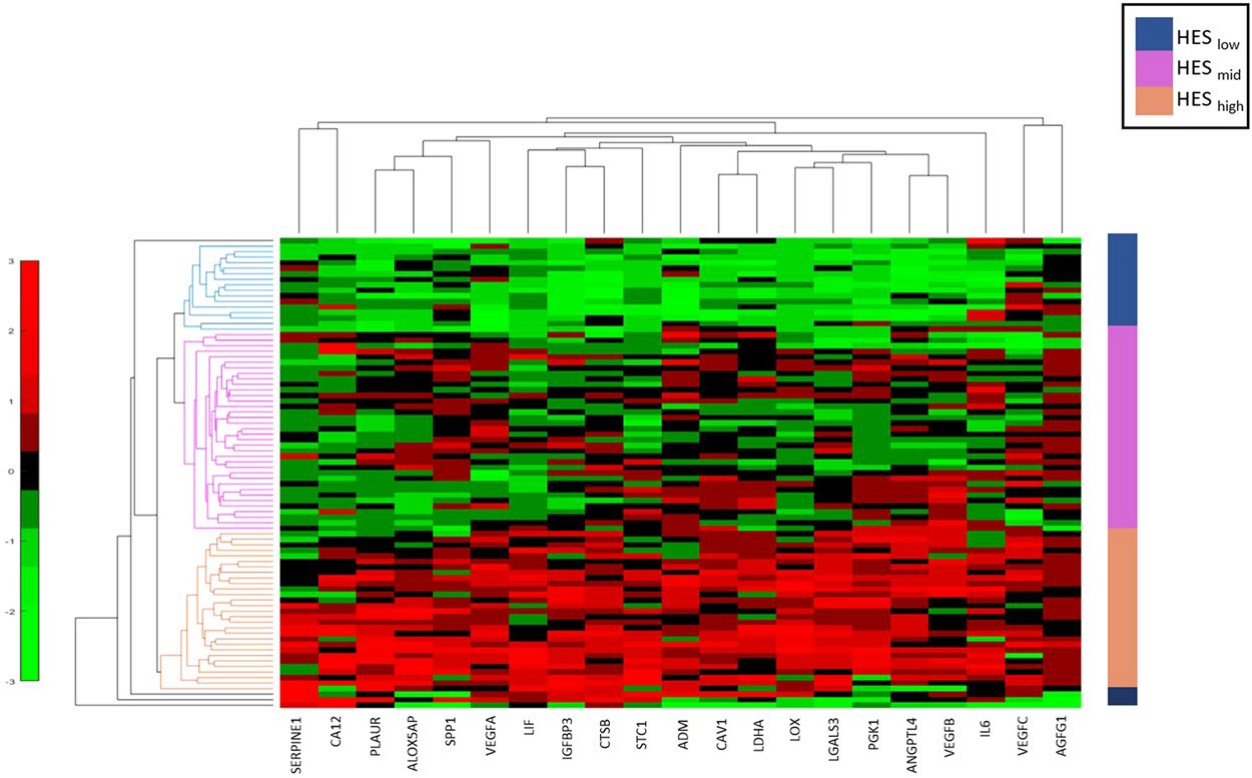
Unsupervised clustering of the RNA seq data from the 21 hypoxia associated genes clustered as *low* hypoxia (HES_*low*_ - shown in navy blue), *medium* hypoxia (HES_*mid*_ - shown in magenta) and *high* hypoxia (HES_*high*_ - shown in orange). The x-axis in the clustergram represents the 21 genes and y-axis represents the patient population of 85 GBM cases.

From previous literature^20^, we identified 21 genes that are implicated in the hypoxia pathway of GBM. Table 1 (Supplementary) lists these 21 genes, and their role in tumor hypoxia. For instance, vascular endothelial growth factor A (VEGFA) gene, an important mediator of angiogenesis, is involved in endothelial cell proliferation and migration^33^. Angiopoietin-like 4 (ANGPTL4) is implicated during hypoxia in GBM and is involved in tumor angiogenesis^34^. Similarly, Galectin-3 (LGALS3) is up-regulated in hypoxic conditions of GBM, which resists cell death, favors cell migration, and thus can be implicated with cancer recurrence^35^. The TCGA GBM mRNA data (Level3 - Affymetrix HT HG U133A) was downloaded from Broad Institute^23^. The genomic data was normalized by Z-score transformation and then used in single-sample Gene Set Enrichment Analysis (ssGSEA) on R platform^18,36^. ssGSEA algorithm captures the biologically significant processes (the hypoxia pathway in our case) and calculates an enrichment score for every patient in the cohort when paired with the 21 hypoxia associated genes^37^. Figure 2 shows the unsupervised clustering of the 21 gene set using euclidean distance, which identified three clusters low, medium, and high, using the HES values. The range of HES for each of the clusters is as follows: HES_*low*_ ≤ 3141.99, 3142 ≤ HES_*Mid*_ ≤ 4102.99, and HES_*High*_ ≥ 4103. HES for each of the 85 GBM patients has been provided in the Supplementary spreadsheet (Sheet 2).

### Experimental Setup

#### Experiment 1: Identifying MRI surrogate markers of tumor hypoxia pathway

A total of 115 patients were split into 85 cases for training and 30 were held-out for validation. In the training phase, 85 patient studies were setup in a 3-fold stratified cross-validation for 50 iterations to create a total of 150 sets such that the samples in the training set were class-balanced. Each set consisted of randomized two-thirds data sampled into training and one-third data used for testing. For every training set, we obtained a mutual information score (captures linear or non-linear mutual dependence between two independent variables) between the radiomic features with the hypoxia enrichment score, and ranked the features based on their mutual information scores during each cross-validation run^21^. Subsequently, we obtained the frequency of occurrence of every feature across 150 runs of cross-validation (50 runs of 3-fold cross-validation). A subset of eight radiomic features with highest frequency of occurrences across the cross-validation runs were retained, for further analysis.

#### Experiment 2: Employing radiomic surrogate markers of tumor hypoxia in predicting patient survival in GBM

The top 8 features (as identified in Experiment 1) were used in a 3-fold cross-validation setup in Experiment 2 for predicting patient survival. Care was taken while setting up the training phase in Experiment 2 to ensure that the same set of training and test sets as used in Experiment 1 were employed, to avoid classifier bias. We then employed a Random Forest (RF) classifier^38^ for survival stratification of patients, as (a) STS versus LTS, (b) STS versus MTS and (c) MTS versus LTS. RF is a commonly used ensemble classifier that combines predictions from several weak decision tree classifiers to generate a more accurate and stable classifier. Treebagger implementation of the RF classifier in MATLAB R2014b was employed, with a total of 50 trees used for training the classifier. Gini impurity was used as the criterion to measure the quality of split. The RF classifier has previously been successfully employed for various biomedical classification applications^12^. Advantages of RF include, (1) ability to integrate a large number of input variables, (2) robustness to noise in the data, and (3) relatively few tuning-parameters. During cross-validation, we further ensured that the data in every fold had equal representation of survival labels. The difference between the 2 groups for survival analysis was assessed by the prediction of random forest classifier, aggregated over the 50 runs within the 3-fold cross-validation.

### Evaluation

Kaplan Meier (KM) survival analysis was used to compare survival times across: (a) STS versus LTS, (b) STS versus MTS and (c) MTS versus LTS, both on training as well as validation set. The horizontal axis on the survival curve shows the time and the vertical axis shows the probability of survival. Any point on the survival curve reflects the probability that a patient in each group would remain alive at that time. Optimal classifier predictions would show maximum separation between the survival curves^12^.

### Statistical Analysis

Survival curves were compared statistically by a Cox proportional hazards model. All statistical analyses were performed using the survival package in R^39,40^. Hazard ratios (HR) were used to quantify the effect of individual feature on survival. Features yielding negative regression coefficients (i.e. low feature values correlated with long term survival) in our Cox Model produce a HR between 0 and 1; features yielding positive regression coefficients (i.e. low feature values correlated with short term survival) produce a HR between 1 and infinity. We also computed Concordance indices (C indices or C statistic) for each of our univariate and multivariate analysis experiments in R. C indices is the fraction of all pairs of subjects whose predicted survival times are correctly ordered (i.e. concordant with actual survival times). C indices = 1 indicates that the model has perfect predictive accuracy, and C indices = 0.5 indicates that the model is not better than random chance.

## Results

### Experiment 1: Identifying MRI surrogate markers of tumor hypoxia pathway

Table 3 lists the top 8 radiomic features that were identified to be most associated with the hypoxia enrichment score using the mutual information feature selection method. The most associated radiomic features included Laws energy (R5R5, E5E5, S5S5) from enhancing tumor and edema capturing ripples, edges and spots. The top 8 features also included entropy, difference variance, and energy features from the Haralick family, which capture structural heterogeneity within the image texture, extracted from edema/nonenhancing and enhancing tumor.

**Table 3:** Top 8 radiomic features identified across MRI scans (Gd-T1w, T2w, FLAIR) that were most associated with the hypoxia enrichment score.

Feature	Tumor Region	Relevance to lesion architectre
Law R5R5	FLAIR Enhancing Tumor	Captures presence of spots, edges, waves and ripples of an image
Law E5E5	Gd-T1w Edema	
Law E5E5	FLAIR Edema	
Law S5S5	T2w Enhancing Tumor	
Information measure of correlation 1 (Haralick)	T2w Necrosis	Captures co-occurrences; quantifies structural heterogeneity
Difference Variance (Haralick)	Gd-T1w Edema	
Energy (Haralick)	FLAIR Enhancing Tumor	
Entropy (Haralick)	Gd-T1w Edema	

**Figure 3:**
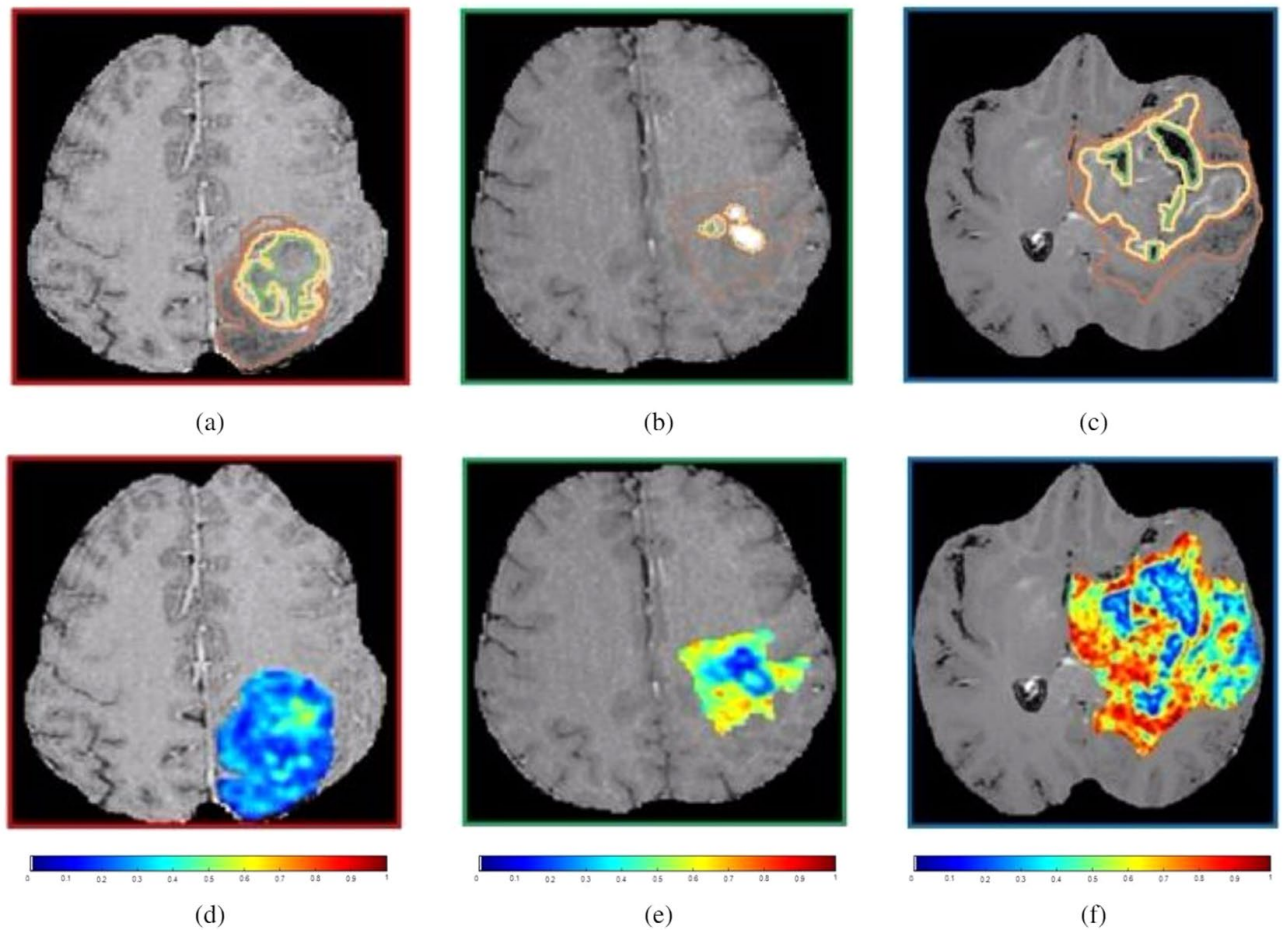
(**a**)–(**c**) show a 2D Gd-T1w MRI slice with expert-annotated necrosis (outlined in green), enhancing tumor (yellow) and edematous regions (brown) in 3 different GBM patients that exhibited low, medium, and high HES respectively. The corresponding Haralick feature map has been overlaid on the manually annotated tumor regions, for HES_*low*_ (**d**), HES_*medium*_ (**e**), and HES_*high*_ (**f**).

Figure 3 shows a single 2D slice of the original Gd-T1w MRI scan with annotations of the 3 tumor sub-compartments (necrosis outlined in green, enhancing tumor in yellow, and edema in brown), and the corresponding Haralick feature map for three different patients with high, medium and low hypoxia enrichment scores, respectively. While the original Gd-T1w MRI scans may not be able to visually capture the underlying tumor heterogeneity of hypoxic tumors, the radiomic features were found to be distinctly different across the varying degrees of hypoxia (low, medium, and high) (Fig. 3). High radiomic feature expressions corresponded to high hypoxia enrichment score (Fig. 3 (f)), while low feature expressions corresponded to low hypoxia enrichment score (Fig. 3 (d)).

### Employing radiomic surrogate markers of tumor hypoxia in predicting patient survival in GBM

**Figure 4:**
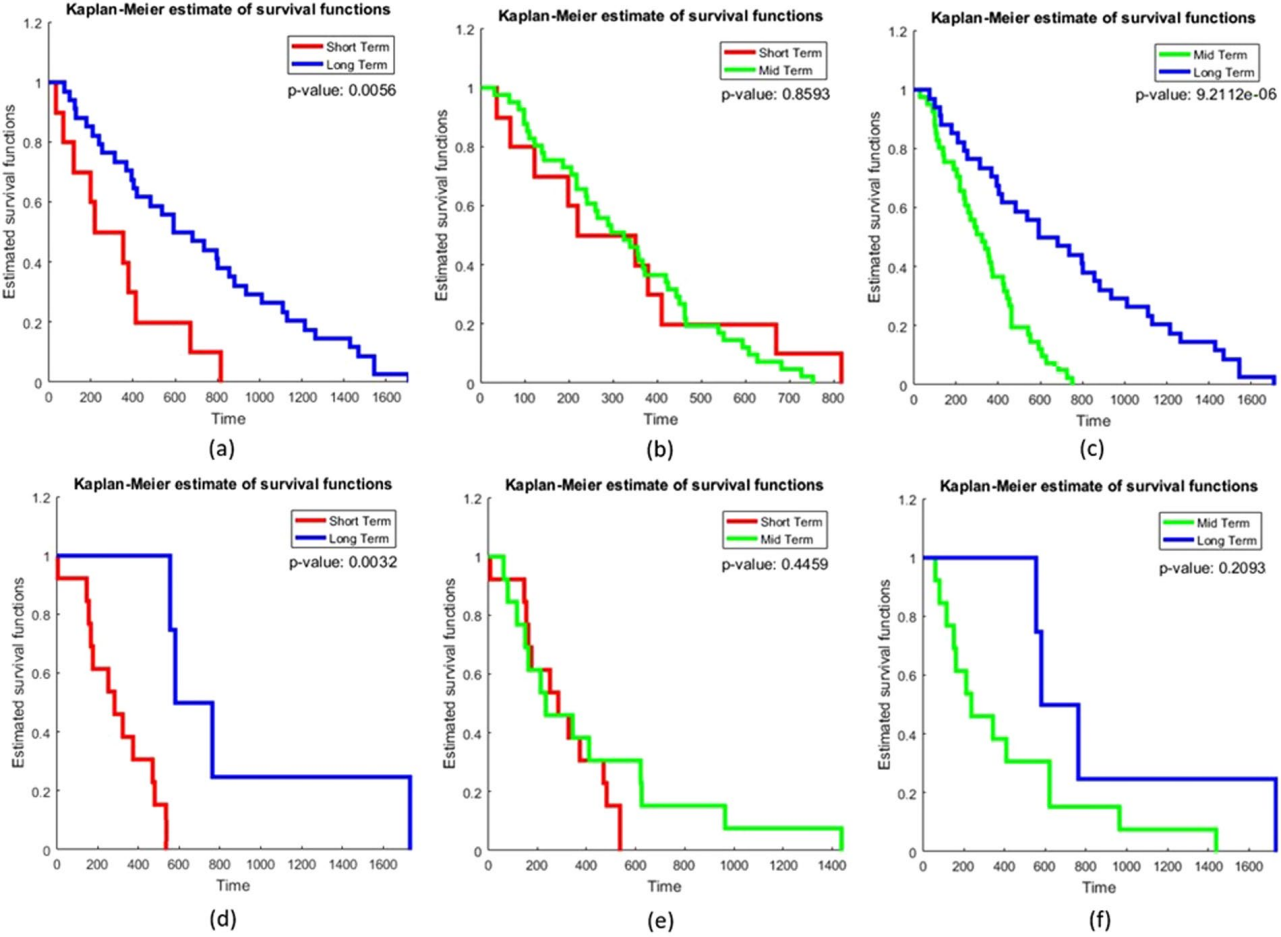
KM curve generated for training (**a**,**b**,**c**) and independent validation set (**d**,**e**,**f**) using the top radiomic features **(a)** short-term (red) and long-term (blue) (p = 0.0056) **(b)** mid-term (green) and short-term (red) (p = 0.8593) and **(c)** mid-term (green) and long-term (blue) GBM survivors (p = 9.21 × 10^−6^) **(d)** short-term (red) and long-term (blue) (p = 0.0032) and (**e**) mid-term (green) and short-term (red) GBM survivors (p = 0.4459) **(f)** mid-term (green) and long-term (blue) (p = 0.2093).

The top 8 radiomic surrogate markers of hypoxia (as identified in Experiment 1) were also found to be significantly associated with survival (Fig. 4). Within the training set, Kaplan-Meier (KM) survival analysis between (a) STS versus LTS, and (b) MTS versus LTS showed a statistically significant separation between the survival curves as quantified via the log-rank test. Figure 4(a) shows the KM curve for patients with STS and LTS (*p* = 0.0056) while 4(b) shows the KM curve generated using the radiomic features between STS versus MTS (*p* = 0.8593), Fig. 4(c) shows the KM curves for MTS versus LTS (*p* = 9.2112×10^−6^). On the validation set, significant differences in KM curves were observed for the short-, versus long-term survival patients (*p* = 0.0032) (Fig. 4 (d)), with a C-index of 0.74. While, the KM curves for mid-term and long-term survival were not found to be statistically significantly different (*p* = 0.2093), the C-index was found to be 0.73. Table 4 lists the hazard ratio and concordance indices for the clinical parameters and combined radiomic features for distinguishing STS versus LTS, and MTS versus LTS. Interestingly, we found that combining clinical and radiomic features improved the concordance index in predicting overall survival in STS versus LTS (C-index = 0.69 and 0.83 on training and validation set respectively) and MTS versus LTS (C-index = 0.7 and 0.81 on training and validation set respectively) as compared to using either clinical features or radiomic features alone (Table 4).

**Table 4:** Hazard ratios of the training set, statistical significance (via *p*-value on training set) and concordance using clinical and radiomic features, obtained from different compartments (edema, necrosis, enhancing tumor) on multi-parametric MRI, for short term versus long term patients and mid term versus long term patients.

Feature	Short term vs Long term				Mid term vs Long term			
	Hazard Ratio	p-value	Concordance Index		Hazard Ratio	p-value	Concordance Index	
			Training set	Validation set			Training set	Validation set
Age	1.03257	0.0103	0.627	0.52	0.977	0.041	0.61	0.51
Gender	1.4219	0.232	0.553	0.64	1.15	0.601	0.52	0.52
KPS	0.98002	0.0571	0.58	0.53	0.98	0.048	0.58	0.62
All radiomic features	0.9722–1.6271	0.0056	**0.65**	**0.74**	0.8443 −1.5108	9.2112 × 10^−6^	**0.69**	**0.73**
All radiomic features and clinical features (age, gender and KPS)	0.9363–1.5923	0.05269	**0.69**	**0.83**	0.8838–1.5604	0.01237	**0.7**	**0.81**

## Discussion

Currently, there is a lack of well validated non-invasive biomarkers that can predict the extent of hypoxia and potentially stratify patients that may be more suited for adjuvant anti-angiogenic treatments. For example, pre-clinical studies have shown that Bevacizumab (a humanized anti-VEGF monoclonal antibody for GBM therapy) attempts to normalize tumor vasculature, reduce hypoxia, and improve drug delivery^41^. Another challenge post-anti VEGF therapy is that patients sometimes fail treatment due to multiple intrinsic properties of the tumor (such as up-regulation of multiple pro-angiogenic factors, enhanced invasion and migration), where a GBM with high levels of hypoxia would indicate that the tumor has found other pathways to proliferate (for example, using ANG2)^7^. Therefore identifying non-invasive methods to monitor the hypoxic micro-environment has significant clinical implications in designing personalized treatment, as well as monitoring response to treatment in GBM patients. In this study, we investigated the relationship between MRI based radiomic features obtained from tumor sub-compartments and the corresponding gene expression data of the hypoxia pathway, as observed on the HES. We found prognostic radiomic features that were capable of distinguishing low, medium, and high levels of hypoxic extent (as defined from the HES), which could potentially also serve as imaging surrogates of overall survival in GBM.

### Radiomic surrogate markers of hypoxia enrichment score

Our study identified Law energy and Haralick features from the edematous/nonenhancing tumor and enhancing tumor region on FLAIR and Gd-T1w MR sequences to be highly associated with the hypoxia enrichment score. We believe that in the enhancing region of GBM, tortuous vessels of hypoxia induced neo-angiogenesis pile up and bulge to manifest as ripples and organization of pseudopalisades in the immediate vicinity of necrosis, evince as hypo-intense rings or spots and therefore are captured by law energy features on FLAIR^42^. This finding is concordant with Diehn *et al*., who found that their genomic expression module of hypoxia correlated with the enhancing region (*p* = 0.012) on Gd-T1w images^20^. Barajas *et al*., also found that enhancing regions of GBM with elevated relative cerebral blood volume (rCBV) were significantly correlated with hypoxia (*p* < 0.02)^43^. Similarly, we identified Haralick features (Entropy, difference variance) from the edematous region to be correlated with hypoxia. These Haralick features might potentially be quantifying the structural heterogeneity in highly hypoxic GBM tumors as observed on Gd-T1w MR sequences. For example, high values of entropy feature reflects high diversity in grey levels of diverse group of pixels (thus quantifying image heterogeneity)^12^.

### Role of hypoxia-radiomic surrogate markers in predicting short-, medium-, long-term survivors of GBM

Imaging features have previously been shown to be prognostic of GBM survival. For example, Zhang *et al*., has demonstrated that the ratio of tumor volume to edema can predict overall survival (*p* < 0.001) in GBM^44^. Similarly, Carrillo *et al*., showed that edema can stratify GBM survival and is associated with poor prognosis in MGMT promoter methylated GBM tumors^45^. By assessing hypoxia using 18F-FMISO PET, Spence *et al*., demonstrated a strong correlation of volume and intensity of hypoxia with poor survival in radiotherapy nave patients of GBM (*p* < 0.002)^46^. Our findings are in consensus with the other research groups that have shown that enhancing region on FLAIR has significant associations with OS (*p* < 0.001) in GBM^47^. Our features also corroborate with our own previous work, where Prasanna *et al*., found that radiomic features from the edematous region on Gd-T1w are predictive of survival in STS and LTS GBM patients (*p* = 1.47 X 10^−5^)^12^. Similarly, in concordance with previous findings^12,47^, the combination of radiomic features with clinical parameters (Table 4) were found to improve prediction of GBM survival, as compared to radiomic features and clinical parameters alone. This suggests the prognostic potential that hypoxia-associated radiomic features offer in conjunction with clinical parameters in characterizing overall survival in GBMs. Lastly, it was observed that the short and medium term survivors were not separable on both the training and validation cohort (Fig. 4), suggesting that these categories may potentially represent a more aggressive radiomic phenotype compared to the long term survival cases.

The work presented in this study did have its limitations. In this study, we limited our analysis to only identifying associations of radiomic features with the hypoxia enrichment score, due to hypoxia’s involvement in chemo-radiation resistance and poor outcomes in GBM. However, the radiomic features that were found to be associated with HES, may also be representative of other carcinogenic signaling pathways, such as tumor infiltration, proliferation, and angiogenesis that are implicated during hypoxia, and are known to contribute to poor outcome. For example, high expression of VEGFA (1 of the 21 genes that contributed to HES), promote repeated cycles of neo-angiogenesis that lead to microvascular hyperplasia, proliferation, and invasion in GBM tumors^48^. An extensive analysis of the association of the radiomic features with the other known pathways contributing to poor outcome (e.g. tumor infiltration, proliferation, and angiogenesis) will be a part of future study. Additionally, the results presented in this paper are preliminary and constrained by a relatively small sample size. A larger independent validation of the radiomic surrogate markers of hypoxia will need to be performed to further validate our preliminary findings. As the data was retrospectively collected from TCIA, another limitation of our study is that only routinely acquired MRI sequences (Gd-T1w, T2w and FLAIR) were used for analysis, and did not employ any advanced imaging (i.e. perfusion, DWI) including PET imaging.

## Conclusion

In this study, we investigated the feasibility of computer-extracted radiomic features from different sub compartments of the tumor on treatment-naïve routine MRI in predicting extent of hypoxia and overall survival in GBM patients. The results suggest that radiomic features from the enhancing and edematous regions appear to be predictive of the extent of hypoxia (as observed using hypoxia enrichment score on mRNA data). The radiomic features on the validation set were also found to be prognostic of LTS vs STS. The identified radiomic features in this work could be used to monitor hypoxia, help determine timeline of treatment resistance, and evaluate the efficacy of anti-angiogenic therapy in GBM and other tumors.

While in this work, we limited the radio-genomic analysis to capturing radiomic imaging phenotypes that were associated with hypoxia pathway, our presented radiogenomic framework, could potentially in the future help identify imaging biomarkers associated with other key pathways such as tumor infiltration, proliferation, and angiogenesis, that are implicated in GBM, and contribute to poor outcomes. Future work will also focus on incorporating complementary imaging parameters obtained from advanced imaging (PET, perfusion, DWI) which may further improve survival prediction using radiomic analysis, while taking into account the extent of resection and subsequent treatment.

## Electronic supplementary material

Supplementary Dataset

Supplementary document
