# Supplementary material for: Radiogenomic analysis of hypoxia pathway is predictive of overall survival in Glioblastoma
Source: Sci Rep. 2018 Jan 8;8:7. doi: 10.1038/s41598-017-18310-0 (PMC5758516; doi:10.1038/s41598-017-18310-0)
Supplement: Supplementary file 2 — Supplementary document [file 41598_2017_18310_MOESM2_ESM.pdf]

Radiogenomic analysis of hypoxia pathway is predictive of overall survival in Glioblastoma

Niha Beig[1,+], Jay Patel[1], Prateek Prasanna[1], Virginia Hill[2], Amit Gupta[3], Ramon Correa[1], Kaustav Bera[1], Salendra Singh[4], Sasan Partovi[3], Vinay Varadan[4], Manmeet Ahluwalia[5], Anant Madabhushi[1], and Pallavi Tiwari[1,+]

[1]Case Western Reserve University, Department of Biomedical Engineering, Cleveland, 44106, USA
[2]Department of Neuroradiology, Imaging Institute, Cleveland Clinic, Cleveland, 44106, USA
[3]University Hospitals of Cleveland, Department of Radiology, Cleveland, 44106, USA
[4]Case Western Reserve University, School of Medicine, Cleveland, 44106, USA
[5]Brain Tumor and Neuro-Oncology Center, Cleveland Clinic, Cleveland, 44106, USA
[+]niha.beig@case.edu; pallavi.tiwari@case.edu

Supplementary file

Preprocessing

For every study, T2w and FLAIR were co-registered with reference to Gd-T1w MRI using 3D affine registration with 12 degrees of freedom encoding rotation, translation, sheer, and scale. The registration was performed using the General Registration (BRIANSFIT) module of 3D Slicer 4.5 (http://www.slicer.org).[1,2] The following parameters were inserted in the registration algorithm: *percentage of samples = 0.002; spline grid size = 14, 10, 12; Interpolation Mode: Linear; maximum iterations = 1,500; maximum step length = 0.05; minimum step length = 0.001; Relaxation factor = 0.5; number of histogram bins = 50; and number of match-points = 10.* To resolve the issue of resolution variability, every MRI slice within a scan was re-sampled to have an uniform pixel spacing of 0.5 x 0.5 mm^2 and was then linearly interpolated to have 3 mm slice thickness using the Resample Image (BRAINS) Module within 3D Slicer. After implementing these registration steps, the 3D Slicer checkerboard module was used to visually inspect the accuracy of registration results.[1] In addition to this, we also computed Jaccard Index (JI)[3] to quantitatively assess the registration accuracy.[4,5] When evaluated on the independent validation set (n=30) from our cohort, the average JI between Gd-T1w and T2w scans was found to be 0.87. Similarly, when the JI was calculated between Gd-T1w and FLAIR, the average was found to be 0.82.

No.	Hypoxia associated genes	Function
1	Urokinase plasminogen activator surface receptor (PLAUR)	Suggestive of regulation of PLAUR expression by HIF-1 representing invasion during hypoxia in GBM.[6]
2	Lysyl oxidase (LOX)	Correlates with upregulation in hypoxic conditions of GBM.[7]Vital for hypoxia-induced metastasis.[8]
3	Leukemia inhibitory factor (LIF)	promotes chemoresistance in colorectal cancers. Hypoxia induces LIF mRNA expression.[9]
4	Stanniocalcin-1 (STC1)	In early stage clear cell renal cell carcinoma, hypoxia induced overexpression of STC1 has shown to be associated with metastasis.[10]
5	Vascular endothelial growth factor-A (VEGFA)	In hypoxic conditions, VEGFA induces HIF that leads to angiogenesis.[11] Associated with poor prognosis.[12]
6	Vascular endothelial growth factor-B (VEGFB)	Belongs to the VEGF family where angiogenesis has been shown in myocardial tissue of an embryo.[13]
7	Vascular endothelial growth factor-C (VEGFC)	Associated with Lymphangiogenesis.[14]
8	Serpin Family E Member 1 (SERPINE1)	This gene is a validated HIF target, which is an inhibitor of fibrinolysis. It has also been shown that in cervical cancer stem cells, activation of this gene leads to degradation of ECM.[15]
9	Insulin-like growth factor-binding protein 3 (IGFBP3)	Under hypoxic conditions, HIF-1α regulates IGFBP3. In newly diagnosed GBM, this gene was found to be prognostic.[16]
10	Adrenomedullin (ADM)	Human ADM gene promoter region contains three HIF-1 binding consensus sites. Upregulated in hypoxic conditions.[17]
11	Angiopoietin-like 4 (ANGPTL4)	In GBM, ANGPTL4 promotes tumor progression and activates extracellular signal-regulated kinase 1/2 (Erk1/2).[18]
12	Phosphoglycerate kinase 1 (PGK1)	In human hepatoma cell lines, it was seen that PGK1 is transcriptionally activated by HIF-1.[19]
13	Lactate dehydrogenase (LDHA)	HIF-1 activates LDHA which is implicated in metabolic responses to intratumoral hypoxia.[20]
14	Caveolin-1 (CAV1)	Direct transcriptional target of HIF, which results in its upregulation leading to proliferation and invasion.[21]
15	Cysteine protease cathepsin B (CTSB)	Dependent on HIF-2α which enhances acute hypoxia.[22]
16	5-lipoxygenase-activating protein gene (ALOX5AP)	In ovarian cancer cells, it was observed that 5-LOX correlated with the density of macrophages in hypoxic areas.[23]
17	Secreted phosphoprotein 1 (SPP1)/ osteopontin (OPN)	In breast cancer, it has been shown that hypoxia driven OPN contributes to tumor growth through angiogenesis.[24]
18	ArfGAPs (AGFG1)	Implicated with HIF in hypoxia.[25]
19	Interleukin 6 (IL6)	In GBM, hypoxia-induced IL6 acts as an initiator of autophagy via pSTAT3 pathway[26]
20	Carbonic anhydrase 12 (CA12)	CA12 is involved in maintaining the extracellular acidic PH in tumors. In cancers, CA12 is overexpressed due to hypoxia.[27]
21	Galectin-3 (LGALS3)	In GBM, the pseudopalisading cells accumulate large amounts of gal-3. Gal3 resists cell death and supports tumor growth in hypoxia.[28]

Table 1. The list of 21 hypoxia associated genes that were used to create the Hypoxia Enrichment Score, and their role in tumor response and survival.

References

1. Fedorov, A. *et al.* 3D Slicer as an Image Computing Platform for the Quantitative Imaging Network. *Magn Reson. Imaging* 1323–41 (2012).
2. H.J, J., G, H. & K., W. BRAINSFit: Mutual Information Registrations of Whole-Brain 3D Images, Using the Insight Toolkit. *Univ. Iow Carver Coll. Medicine, Dep. Psychiatry NeuroImaging Cent.* (2007).
3. Jaccard, P. Etude comparative de la distribution florale dans une portion des Alpes et du Jura. *Bull. de la Soc. Vaudoise des Sci. Naturelles* (1901).
4. Ou, Y., Akbari, H., Bilello, M., Da, X. & Davatzikos, C. Comparative Evaluation of Registration Algorithms in Different Brain Databases With Varying Difficulty: Results and Insights. *IEEE Transactions on Med. Imaging* (2014).
5. A, K. *et al.* Evaluation of 14 nonlinear deformation algorithms applied to human brain MRI registration. *Neuroimage* (2009).
6. Persson, M. *et al.* uPAR as a Potential New Imaging Biomarker in Glioblastoma. *J. Nucl. Medicine* (2015).
7. Bar, E. E., Lin, A., Mahairaki, V., Matsui, W. & Eberhart, C. G. Hypoxia Increases the Expression of Stem-Cell Markers and Promotes Clonogenicity in Glioblastoma Neurospheres. *The Am. J. Pathol.* (2010).
8. Mimeault, M. & Batra, S. K. Hypoxia-inducing factors as master regulators of stemness properties and altered metabolism of cancer- and metastasis-initiating cells. *J Cell Mol Med* (2013).
9. Wu, L. *et al.* HIF-2α mediates hypoxia-induced LIF expression in human colorectal cancer cells. *Oncotarget* (2015).
10. Ma, X. *et al.* Hypoxia-induced overexpression of stanniocalcin-1 is associated with the metastasis of early stage clear cell renal cell carcinoma. *J. Transl. Medicine* (2015).
11. Harmey, J. *VEGF and Cancer* (2004).
12. Flynn, J. R. *et al.* Hypoxia-regulated protein expression, patient characteristics, and preoperative imaging as predictors of survival in adults with glioblastoma multiforme. *Cancer* (2008).
13. Claesson-Welsh, L. VEGF-B Taken to Our Hearts: Specific Effect of VEGF-B in Myocardial Ischemia. *Arter. Thromb. Vasc. Biol.* (2008). DOI 10.1161/ATVBAHA.108.170878.
14. Jeltsch, M., Tammela, T., Alitalo, K. & Wilting, J. Genesis and pathogenesis of lymphatic vessels. *Cell Tissue Res.* (2003).
15. Sato, M. *et al.* Decreased expression of the plasminogen activator inhibitor type 1 is involved in degradation of extracellular matrix surrounding cervical cancer stem cells. *Int. J. Oncol.* (2015).
16. Santosh, V. *et al.* Grade-Specific Expression of Insulin-like Growth Factor–Binding Proteins-2, -3, and -5 in Astrocytomas: IGFBP-3 Emerges as a Strong Predictor of Survival in Patients with Newly Diagnosed Glioblastoma. *Cancer Epidemiol. Biomarkers Prev.* (2010).
17. Garayo, M. *et al.* Hypoxia-inducible factor-1 (HIF-1) up-regulates adrenomedullin expression in human tumor cell lines during oxygen deprivation: a possible promotion mechanism of carcinogenesis. *Mol. Endocrinol.* (2000).
18. Brunckhorst, M., Wang, H., Lu, R. & Yu, Q. Angiopoietin-4 promotes glioblastoma progression by enhancing tumor cell viability and angiogenesis. *Cancer Res.* (2010).
19. Kress, S. *et al.* Expression of hypoxia-inducible genes in tumor cells. *J Cancer Res Clin Oncol* (1998).
20. Semenza, G. L. HIF-1 mediates metabolic responses to intratumoral hypoxia and oncogenic mutations. *The J. Clin. Investig.* (2013).
21. Wang, Y. *et al.* Hypoxia promotes ligand-independent EGF receptor signaling via hypoxia-inducible factor–mediated upregulation of caveolin-1. *Proc Natl Acad Sci* (2012).
22. Fuhrmanna, D. C. *et al.* Inactivation of Tristetraprolin in Chronic Hypoxia Provokes the Expression of Cathepsin B. *Mol. Cell. Biol.* (2014).
23. Wen, Z. *et al.* Increased metabolites of 5-lipoxygenase from hypoxic ovarian cancer cells promote tumor-associated macrophage infiltration. *Oncogene* (2014).
24. Raja, R. *et al.* Hypoxia-driven osteopontin contributes to breast tumor growth through modulation of HIF1α-mediated VEGF-dependent angiogenesis. *Oncogene* (2014).
25. Nekhai, S., Kumari, N. & Dhawan, S. Role of cellular iron and oxygen in the regulation of HIV-1 infection. *Futur. Virol.* (2013).

26. Xue, H. *et al.* A novel tumor-promoting mechanism of IL6 and the therapeutic efficacy of tocilizumab: Hypoxia-induced IL6 is a potent autophagy initiator in glioblastoma via the p-STAT3-MIR155-3p-CREBRF pathway. *Autophagy* (2016).

27. Ivanov, S. *et al.* Expression of Hypoxia-Inducible Cell-Surface Transmembrane Carbonic Anhydrases in Human Cancer. *The Am. J. Pathol.* (2001).

28. Ikemori, R. Y. *et al.* Galectin-3 Up-Regulation in Hypoxic and Nutrient Deprived Microenvironments Promotes Cell Survival. *PLOS One* (2014).
